# Research Progress of Exosomal Non-Coding RNAs in Cardiac Remodeling

**DOI:** 10.7150/ijms.83808

**Published:** 2023-09-11

**Authors:** Yang Liu, Xing Lyu, Shengyu Tan, Xiangyu Zhang

**Affiliations:** 1 Department of Geriatrics, The Second Xiangya Hospital, Central South University, Changsha, Hunan 410011, China.; 2 Department of Clinical laboratory Medicine, The Second Xiangya Hospital, Central South University, Changsha, Hunan 410011, China.; 3 Hunan Clinical Medical Research Center for Geriatric Syndrome, Changsha, Hunan 410011, China.

## Abstract

Exosomes are vesicles with a size range of 50 to 200 nm and released by different cells, which are essential for the exchange of information between cells. They have attracted a lot of interest from medical researchers. Exosomal non-coding RNAs play an important part in pathological cardiac remodelings, such as cardiomyocyte hypertrophy, cardiomyocyte apoptosis, and cardiac fibrosis. This review summarizes the origins and functions of exosomes, the role of exosomal non-coding RNAs in the process of pathological cardiac remodeling, as well as their theoretical basis for clinical application.

## Background

Cardiovascular diseases (CVDs) continue to be the main causes of morbidity and mortality globally. As CVDs worsen, irreversible cardiac remodeling will occur, ultimately leading to heart failure (HF) 1. Early cardiac remodeling is originally thought to be an active compensatory alteration of the body in response to increased stress, recent research showed it is directly associated with the morbidity and mortality of CVDs 2. Human cardiac tissues are composed of various cell types including cardiomyocytes, cardiac fibroblasts, endothelial cells (ECs), smooth muscle cells, and a few cardiac stem cells. Additionally, there are a number of transitory cells that are linked to CVDs, including mast cells, macrophages, and lymphocytes. The presence and interation of several cardiac cells form a complex intercellular network comprised of numerous signaling pathways, regulating cell-cell connections and/or cell-extracellular matrix interactions, as well as autocrine, paracrine, endocrine, etc 3, 4.

Exosomes have recently been identified as having an important role in cellular communication. Apart from abundant physiological and signaling tasks, exosomes are necessary for removing cellular wastes 5. Exosomes are released by various type of cells, including stem cells, ECs, fibroblast cells, and even tumor cells. Through the intercellular connections, multicellular organisms remain homeostatic maintenance 6. Exosomes are round-shaped particles that form by the inward budding and fission of late endosomal vesicle membranes and have been found to contain a wide range of biomolecules, such as proteins, cytokines, messenger RNAs (mRNAs), and non-coding RNAs (ncRNAs) 7. Carrying these informative molecules, exosomes are considered to serve as key cell-to-cell messengers that regulate homeostasis and have an impact on the pathophysiology of several diseases, including CVDs 4.

Based on the structure, length of nucleotide and function, ncRNAs are divided into microRNAs (miRNAs or miRs), long noncoding RNAs (lncRNAs), circular noncoding RNAs (circRNAs), transfer RNAs, ribosomal RNAs, small nuclear RNAs, small nucleolar RNAs, guide RNAs, piwi-interacting RNAs, and small interfering RNAs 8. MiRNAs are short ncRNAs with a length of 19-25 nucleotides which are highly conserved and regulate gene expression. By binding to the 3' untranslated region (3'-UTR) of target mRNA, it inhibits the translation of mRNA or promotes its degradation. Many miRNAs control the same mRNA, and one miRNA is capable of controlling multiple mRNAs. MiRNAs are involve in the process of various diseases, including CVDs 9. LncRNAs are defined as ncRNAs of over 200 nucleotides. According to the position of lncRNAs relative to protein-coding genes, they can be divided into five groups: sense, antisense, bidirectional, intergenic and intronic. LncRNAs have the ability to attach to proteins, RNA, and DNA and regulate their functions. Endogenous cavernous RNAs, namely competing endogenous RNAs (ceRNAs), can interact with miRNAs to affect the expression of mRNAs 10. CircRNAs are a class of endogenous ncRNAs that form continuous closed loops with strong tissue specificity, which are mainly derived from exons and introns, and generated from back-splicing of premRNA. Compared with linear RNAs, circRNAs are characterized by the absence of free 3' or 5' ends. CircRNAs act as sponges of miRNAs, regulating selective splicing and parental gene expression. CircRNAs appear to be implicated in the formation and development of CVDs and tumors, and have the possibility to become new targets of disease treatment and new biomarkers for clinical diagnosis and prognosis 11.

As a stable carrier of information communication between cells, exosomal ncRNAs can precisely regulate cellular signaling pathways and participate in multiple pathophysiological processes such as vascular remodeling, myocardial ischemia, cardiac hypertrophy, and inflammatory immune response 4. Regarding these significant roles of ncRNAs in regulating compensatory and non-compensatory cardiac remodeling, along with the roles of exosomes as carriers of ncRNAs, exosomal ncRNAs are considered a promising new therapeutic target of CVDs 12. This review highlights the function of various exosomal ncRNA in the pathogenesis of CVDs as well as advanced therapeutic procedures.

## Introduction of exosomes

### The source of exosomes

**Figure 1:**
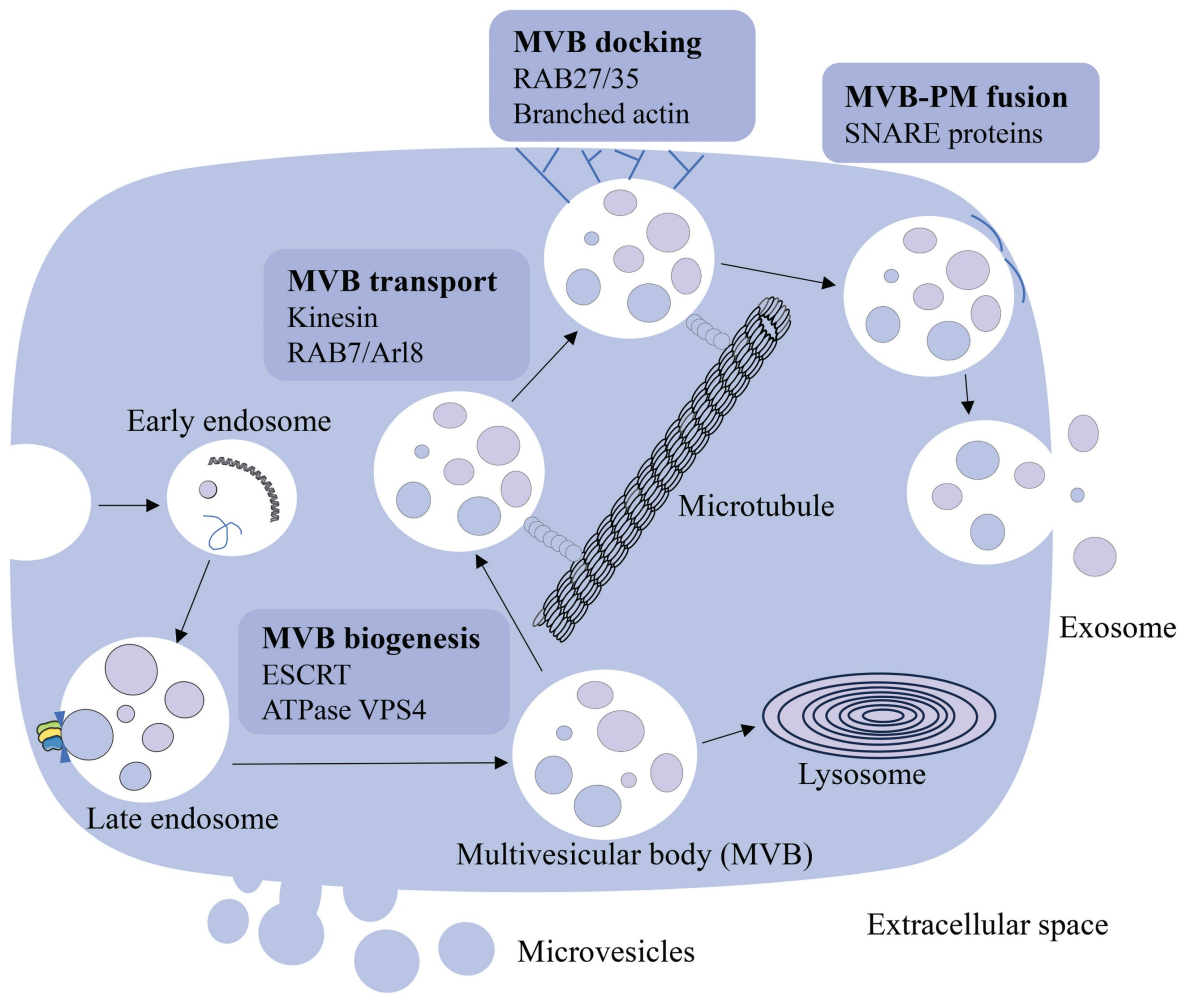
The source and formation of exosomes. Early endosomes are generated by the plasma membrane budding inward and mature into the late endosome. Late endosome becomes multivesicular body (MVB) through regulation of ESCRT and ATPase VPS4. MVBs release exosomes into the extracellular space through MVB transport, docking and MVB-PM fusion processes, or are degraded by the lysosome.

Cells communicate with each other and work as a collective *in vivo* through direct interactions and soluble molecules such as cytokines. Pan et al. first described the vesicles released by sheep reticulocytes as exosomes in 1987, and later proved their presence using electron microscopy 13. In early reports, extracellular vesicles, including exosomes, were considered as cellular waste 14, 15. Recent studies have shown that extracellular vesicles are also involved in intercellular communication. Exosomes are membrane-bound vesicles with sizes ranging from 50 to 200 nm and are found in virtually all biological fluids, including blood 16. They are created by the early endosomes' limiting membranes budding inward, and mature into multivesicular bodies (MVBs) 16-18. Endosomal sorting complexes required for transport (ESCRT) regulate MVBs (including exosomes) synthesis and release 19, 20. This process is initiated by ESCRT-0, which recognizes and retains specific proteins in the late endosomal membrane. After ESCRT-I/II triggers the degradation of the limiting membrane into the MVB cavity, ESCRT-III forms a spiral structure, constricts the budding neck and ATPase VPS4 drives the membrane scission 21-23. MVBs are involved in the endocytosis and transportation of the cell's material 24, eventually either delivered to the lysosome and degraded with all their components, or fuse with the cell membrane and discharge the contents (including exosomes) into the extracellular environment 17, 25. MVBs destined for exocytosis are transported to the plasma membrane along microtubules by the molecular motor kinesin. MVBs transport is mediated by a variety of kinesin isoforms and can be regulated by Arl8- and RAB7-dependent protein complexes 26, 27. MVB docking to the plasma membrane is regulated by RABs, such as RAB27 and RAB35, and the docking site is formed by stabilizing the branched actin filaments 28-30. After transport and docking to the plasma membrane, secreted MVB fuses with the plasma membrane via soluble N-ethylmaleimide-sensitive component attachment protein receptor (SNARE). Humans have a variety of SNARE proteins that locate in different intracellular membranes and mediate the fusion of cell compartments by forming different SNARE complexes 31, 32. When MVBs fuse with the plasma membrane, exosomes are released into the extracellular space, where they interact with the extracellular matrix to affect cells in the microenvironment, and also can enter the circulation through lymph or blood (Figure 1). The elements that influence the fate of a certain MVB are not fully known 18, however, it has been shown it is associated with the level of cholesterol in MVB. Specifically, cholesterol rich vesicles were secreted, whereas cholesterol deficient vesicles were directed to the lysosome for degradation 33.

### Formation and role of exosomes

Exosomes are released into the extracellular space by most cells after fusion with the plasma membrane. The main components of exosome membranes are lipids and proteins, which are enriched with lipid rafts, tetraspanins such as CD9 and CD63, and membrane transport proteins 16, 25, 34, 35. Because the formation of exosomal and the transport of MVB are regulated by ESCRT proteins, these proteins and their accessory proteins (Alix, TSG101, HSC70, and HSP90β) can be found in exosomes regardless of the cell type 36. In addition to lipids and proteins, various nucleic acids, such as mRNAs, miRNAs, circRNAs, and other ncRNAs, have recently been discovered in exosomes 37, 38. When exosomes circulate, these exosomal RNAs can be taken up by neighboring or distant cells and modulate recipient cells. Exosomes have received increased attention because of the discovery of their role in cell-to-cell genetic exchange.

Initially, researchers thought that exosomes were vectors during reticulocyte maturation. Subsequently, some studies have revealed that exosomes play an important role in variety of pathophysiological processes, such as mediating information communication between cells, participating in immune response, signal transduction, and waste removal 39. Recently, studies have shown that exosomes deliver proteins, RNAs, and other contents to the recipient cells by autocrine, paracrine, or endocrine and then affect the biological functions of the recipient cells. The way that the exosomes bind to recipient cells include: releasing active components near the recipient cells; binding to surface-specific receptors of the target cell; fusion with plasma membrane and endocytosis of receptor cells 40. Exosomal ncRNAs offer some benefits in the diagnosis and treatment of diseases.

## Exosomes in cardiac remodeling

It is considered that cardiac stress can lead to fibroblast proliferation, extracellular matrix protein secretion, cardiomyocyte hypertrophy, cardiomyocyte death, and the production of proinflammatory cytokines 4. Notably, 28% cells are cardiomyocytes and 70% is cardiac fibroblasts in human heart 41. Therefore, cardiac myocytes and cardiac fibroblasts are the main determinants of cardiac remodeling. We concentrate principally on two forms of cardiac remodeling in this review: reactive interstitial remodeling and focal or diffuse replacement remodeling. Reactive interstitial remodeling is characterized by increased collagen production and diffused collagen deposition without a loss of myocytes. This type of remodeling happens gradually with increased pressure and/or volume loads, such as in hypertension and aortic stenosis. Eliminating the harmful stimuli or using specific therapy may be possible to reverse the condition. Diffuse or focal replacement remodeling is caused by the death of cardiomyocytes and usually occurs after myocardial infarction (MI). Since the affected myocardium does not survive and the contractile abilities are unable to recover, this type of remodeling is irreversible. We review the role of exosomal ncRNAs in cardiac remodeling to provide fresh perspectives for the mechanism and treatment of cardiac remodeling.

### Exosomal miRNAs in cardiac remodeling

**Figure 2:**
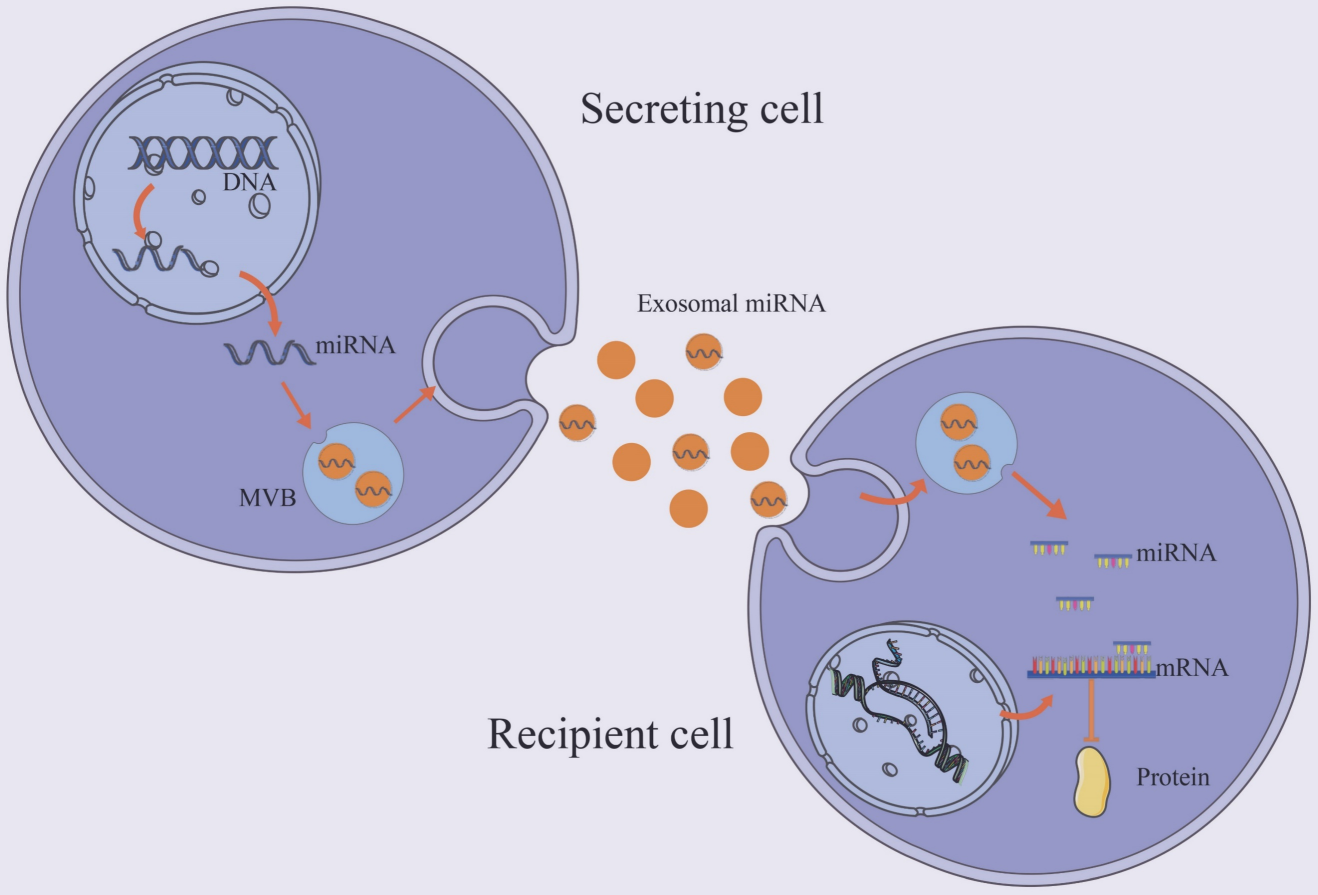
Trafficking and biological function of exosomal miRNAs. The majority of miRNAs are coded by their own genes and transported to the cytoplasm and enters the exosome. Exosomal miRNA regulates post-transcriptional gene expression after taken up by a neighboring or distant cell. It binds the target mRNA according to partial sequence complementarity. By forming a complex with specific proteins, miRNAs inhibit protein synthesis.

MiRNAs are small endogenous oligonucleotides of 21-25 nucleotides, which are essential for regulation of post-transcriptional gene by attaching to or inhibiting target mRNAs 42. The syntheses of majority miRNAs are encoded by their own genes, transcribed by RNA polymerase II. A small number of miRNA sequences, on the other hand, are encoded in other RNA molecules. Exosomal miRNAs enter the target cell and, based on partial sequence complementarity, bind the target mRNA 43 (Figure 2). Because miRNA regulation is sequence-dependent, one mRNA can be silenced by multiple miRNAs, and one miRNA can target multiple mRNAs. Exosomes miRNAs have been found to play a a critical role in cardiac pathology 44, 45.

Exosomes contain a large number of miRNAs, which are diversified from cell types and in response to disease. Mir-21 levels are higher in failing hearts, suggesting a possible role in cardiac remodeling 46. However, inhibiting or overexpressing miR-21 in cardiomyocytes has no effect on the morphology or size of cardiomyocytes 47. Further evidence suggests that miR-21 levels are much higher in fibroblast-derived exosomes than in cells, implying that miR-21 is packed into exosomes 48. During cardiac stress, exosomal miR-21-3p from angiotensin II (Ang II)-stimulated cardiac fibroblasts induced cardiomyocyte hypertrophy by inhibiting sorbin and SH3 domain-containing protein 2 (SORBS2), PDZ and LIM domain 5 (PDLIM5). In cardiomyocytes, silencing or inhibiting miR-21-3p attenuated hypertrophy. Thus, exosomal miR-21, rather than intracelluar miR-21, plays an important role in the transfer of pro-hypertrophy information from cardiac fibroblasts to cardiomyocytes 48. Another study found that Ang II induced exosomes secreted by cardiac myofibroblasts can also enhance the activity of the renin-angiotensin system in cardiomyocytes and promote the hypertrophy of cardiomyocytes by activating mitogen-activated protein kinases (MAPKs) and Akt; exosome suppressor could effectively reduce this pathological process 49. Exosomes derived from macrophages with enriched miR-155 can promote cardiac fibrosis and hypertrophy in uremic mice by activating the pro-hypertrophic pathway FOXO3a 50. MiRNA-enriched exosomes derived from TNF-treated fibroblasts not only inhibited the Nrf2/ARE signaling pathway, but also promoted the expression of cardiac hypertrophy-related genes, implying that these exosomes can inhibit Nrf2 translation and subsequent transcription of downstream targeting genes that contribute to cardiac hypertrophy 51. Cardiac progenitor cells (CPC) -derived exosomal miR-21 inhibited cardiomyocyte's apoptosis pathway through downregulating programmed cell death 4 (PDCD4) 52. Exosomal miRNA-24 derived from rat plasma were also found to have antiapoptotic effects after remote ischemic preconditioning 53. In addition, exosomal miR-19a-3p, miRNA-214, miR-146a, miR-210 and miR-338 were associated with cardiomyocyte apoptosis 54-58.

Exosomal miRNAs can aggravate or reduce cardiac fibrosis, inflammation, and scar formation by regulating gene expression. Macrophage-derived miR-155 as a paracrine regulator can promote cardiac fibroblast proliferation and collagen production 59. Exosomal miR-24-3p derived from mesenchymal stem cells (MSCs) can protect myocardial function after MI, reduce inflammation and fibrosis, inhibit cardiac fibroblast differentiation, promote cardiomyocyte proliferation, and reduce apoptosis 53, 60. Exosomal miR-132 has the ability to reduce scarring, infarct zone size, inflammation and myofibroblast proliferation 61. MiR-378 exosomes from cardiac myocytes entered cardiac fibroblasts, resulting in increased miR-378 levels, which can inhibit the myocardial fibrosis by regulating p38 MAPK signaling pathways 62. Increased exosomal miR-146a-5p expression from cardiosphere-derived cells (CDCs) was related to a decrease in myocardial fibrosis via inhibition of proinflammatory cytokines and transcripts 63. One study had shown that the levels of serum exosomal miR-425 and miR-744 were reduced in 31 patients with HF and Ang II treated cardiac fibroblasts; further investigation revealed these two miRNAs suppressed angiotensin-induced collagen and cellulose synthesis, as well as myocardial remodeling by targeting transforming growth factor-β (TGF-β) 64. For myocardial fibrosis caused by acute MI (AMI), previous studies have shown that MSC-derived exosome miR-22 can inhibit myocardial cell fibrosis by binding methylated CpG binding protein 2 and reduce the degree of myocardial necrosis and fibrosis 65. In addition, increased expression of miR-24 and miR-29 and decreased expression of miR-15, miR-21, miR-34, miR-130, and miR-378 in bone marrow mesenchymal stem cell-derived exosomes inhibited myocardial fibrosis and improved cardiac function in mice after MI 66. Exosomal miR-27a, miR-28, and miR-34a in a rat HF model derived from cardiac fibroblasts contribute to dysregulation of Nrf2/ARE signaling pathway, which resulted in myocardial dysfunction and heart inflammation 51. Exosomal miR-208a was discovered to cause and exacerbate cardiac fibrosis 67.

**Figure 3:**
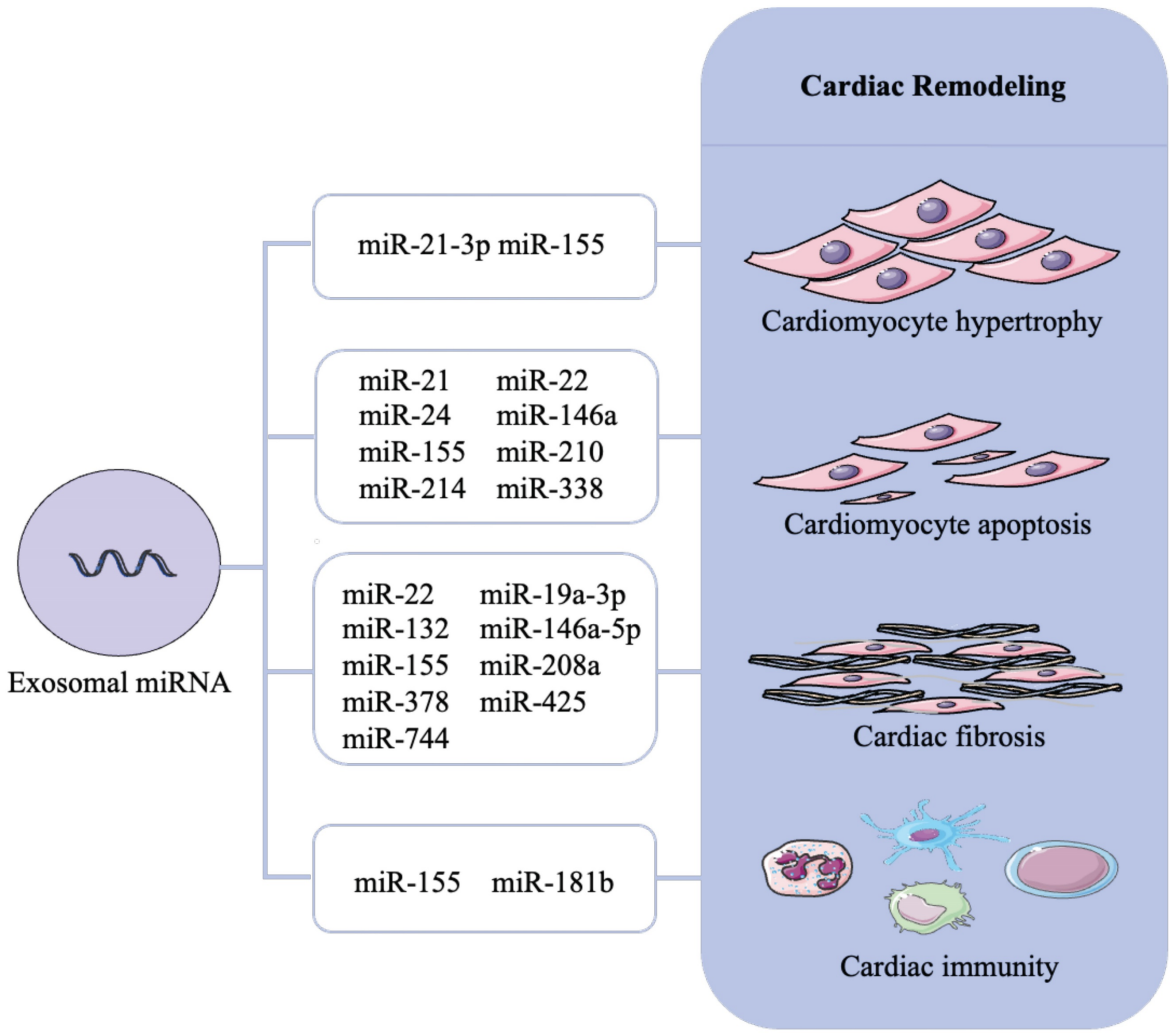
Role of exosomal miRNAs in cardiac remodeling. Cardiac remodeling mainly includes cardiomyocyte hypertrophy, cardiomyocyte apoptosis, cardiac fibrosis and cardiac immunity. Different exosomal miRNAs cause cardiac remodeling through different mechanisms and even multiple mechanisms.

Reported exosomal miRNA that associated with cardiac remodeling are presented in Figure 3 and Table 1.

### Exosomal lncRNAs in cardiac remodeling

LncRNA has been discovered for years, its significance and universality are well known due to the development of high throughput sequencing technologies and the completion of large human genome sequencing projects 68. Accumulating evidence suggests that lncRNAs play a role in the regulation of CVDs and pathological remodeling 69, 70. Despite their large size, some lncRNAs can be transported through extracellular vesicles (EVs), where they are protected by RNase without being degraded 71, 72. Exosomes could deliver cardioprotective and anti-fibrotic lncRNA to the desired site with specificity 73.

In a study, exosomal lncRNAs of AMI patients and controls were sequenced, a total of 518 lncRNAs were found to be differentially expressed over a two-fold change, and circulating exosomal lncRNAs ENST00000556899.1 and ENST00000575985.1 were noticeably elevated in AMI patients compared with controls 74. Hypoxia upregulated the level of exosomal AK139128 from cardiomyocytes and exacerbated MI in the rat model. Moreover, exosomal AK139128 stimulated apoptosis and inhibited proliferation, migration, and invasion of cardiac fibroblasts 75. The exosomal lncRNA KLF3-AS1 secreted by MSCs can regulate Sirt1 to suppress cardiomyocyte viability, apoptosis, and pyroptosis by acting as a ceRNA to sponge miR-138-5p; transfection of miR-138-5p inhibitor and incubation of KLF3-AS1 exosome contribute to reduce pyroptosis both *in vivo* and *in vitro*, whereas sh-Sirt1 transfection may reverse the protective effect of exosomal KLF3-AS1 on hypoxia cardiomyocytes 76. Exosomal lncRNA MALAT1 prevented aging-induced cardiac dysfunction and lncRNA H19 protected cardiomyocytes by enhancing neovascularization 77, 78. Exosomal lncRNA ZFAS1 induced heart fibrosis via the wnt4/β-catenin signal pathway 79.

Currently, there are few studies in exosomal lncRNA and cardiac remodeling, further experiments are needed to find out its potential mechanism. Reported exosomal lncRNA that associated with cardiac remodeling are presented in Table 2.

### Exosomal circRNAs in cardiac remodeling

CircRNA is a type of ncRNA that is produced during the backsplicing of exons or from lariat introns 80, 81. In contrast to linear RNAs, the 3' and 5' ends of a circRNA strand are linked together by covalent bonds to form a stable and conserved circular structure. CircRNAs are mainly present in the cytoplasm, but also found in the nucleus and EVs.

CircRNAs may be vital for cardiac disease progression and treatment. By microarray assay and quantitative PCR, it was found that 29 circRNAs were up-regulated and 34 were down-regulated in a post-MI model of mice. The expression of circRNA-010567 was up-regulated and predicted by bioinformatics software that it could sponge miR-141 82. In cardiac fibroblasts, silencing circRNA-010567 could increase miR-141 expression, decrease TGF-β1 expression, and inhibit collagen I, collagen III, and SMA expression. CircRNA-010567/miR-141/TGF-β1 axis is important in the diabetic myocardial fibrosis mouse model 83.

Interestingly, through RNA sequence analysis, exosomal circRNAs were found to be more plentiful and stable than those weren't encapsulated in exosomes 84. Next-generation sequencing of exosomes from HF patients and a healthy control group found that the exosomal hsa circ-0097435 was elevated in HF patients 85. Human umbilical cord mesenchymal stem cells (UMSCs) derived exosomes can improve the cardiac function after MI by delivering circ-0001273. Compared with the circ-0001273-exosome-treated rats, the cardiac structure and function was remarkably deteriorative in the si-circ-0001273-exosome-treated group; circ-0001273 can remarkably inhibit the occurrence of myocardial cell apoptosis and improve cardiac function in the ischemic environment 86.

Research about the role of exosomal circRNA in cardiac remodeling is in early stage. Reported exosomal circRNA that associated with cardiac remodeling are presented in Table 2.

### Exosomes mediated cardiac immunity in cardiac remodeling

Neurohumoral factors are the major stimulating factors in cardiac remodeling. Various inflammatory cytokines and immune cells are involved in this process, including interleukin 6 (IL-6), macrophages, monocytes, and T cells. Exosomes can encapsulate inflammatory cytokines and act on other cells to participate in the inflammatory process of cardiac remodeling. Exosomal miRNA-181b secreted by cardiomyocytes can inhibit the invasion process of macrophages and mediate immune response 87. In the exosomes secreted by macrophages, miRNA-155 was found to target cardiac fibroblasts and promoted the inflammatory response of fibroblasts 59. Exosomes secreted by hypoxic dendritic cells could upregulate the expression of inflammatory factor IFN-γ and TNF-α in CD4+T cells, and thus promote the activation of T cells 88. Another study found that the plasma exosomes were increased in HF patients compared with the control group, and the exosomal miRNA promoted inflammatory responses through the TLR9-NF-κB signaling pathway 89. These results suggest that exosomal ncRNA can act as vectors to regulate the expression of inflammatory factors and participate in the inflammatory response in the process of cardiac remodeling.

## Conclusion

Given the global aging of populations and the associated rise in HF prevalence, there is an urgent need to develop therapeutic interventions that prevent cardiac remodeling. Since the discovery of exosomal ncRNAs in the past decades, their role in the process of cardiac remodeling development has received increasing attention. Cellular and animal experiments have confirmed that exosomal ncRNAs can aggravate or alleviate cardiac remodeling. In clinical experiments, there are only some exosomal miRNAs was used for the diagnosis of cardiac remodeling 90-94 (Table 3), no ncRNA has been used as clinical drug yet. The research on exosomal lncRNA and circRNA in cardiac remodeling is limited. Further and in-depth research on the mechanism and role of exosomal ncRNAs can help to identify new interacting molecules and signal transduction pathways for cardiac remodeling, providing new ideas and methods for diagnosis and treatment for CVDs.

## Figures and Tables

**Table 1:** Exsomal miRNA which are identified to modulate cardiac remodeling

Exsomal cargo	Communication	Targets	Functions	References
miRNAs				
miR-19a-3p	Between endothelial cells and heart	ND	Promotes angiogenesis, decrease myocardial fibrosis, and improve left ventricular function	54
miR-21-3p	Between cardiomyocytes and cardiac fibroblasts	SORBS2, PDLIM5	Promotes cardiomyocytes hypertrophy	48
miR-21	Between CPCs and cardiomyocytes	PDCD4	Reduces cardiomyocytes apoptosis	52
miR-24	Between plasma and cardiomyocytes	ND	Reduces cardiomyocytes apoptosis	53
miR-146a	Between cardiosphere-derived cells and cardiomyocytes	ND	Reduces apoptosis and promote proliferation of cardiomyocytes	56
miR-155	Between macrophages and cardiomyocytes	FOXO3a	Promotes cardiomyocytes hypertrophy and apoptosis	50
miR-210	Between MSCs and cardiomyocytes	ND	Reduces cardiomyocytes apoptosis	57
miR-214	Between adipose cells and cardiomyocytes	ND	Reduces cardiomyocytes apoptosis	55
miR-338	Between MSCs and cardiomyocytes	MAP3K2	Reduces cardiomyocytes apoptosis	58
miR-155	Between macrophages and cardiac fibroblasts	Son of Sevenless 1	Promotes cardiac fibroblast proliferation and and collagen production	59
miR-24-3p	Between MSCs and heart	ND	Inhibits cardiac fibroblasts differentiation, promotes cardiomyocytes proliferation	53, 60
miR-132	Between CPC and heart	RasGAP-p120	Inhibits cardiac fibrosis	61
miR-378	Between cardiomyocytes and cardiac fibroblasts	MKK6	Inhibits cardiac fibrosis	62
miR-425	Between plasma and heart	TGFβ1	Inhibits cardiac fibrosis	64
miR-744	Between plasma and heart	TGFβ1	Inhibits cardiac fibrosis	64
miR-146a-5p	Between CDCs and heart	ND	Inhibits cardiac fibrosis	63
miR-22	Between MSCs and heart	Mecp2	Inhibits cardiac fibrosis and necrosis	65
miR-208a	Between cardiomyocytes and cardiac fibroblasts	Dyrk2	Increases fibroblast proliferation and differentiation	67

“ND” (Not Determined) indicates that a target has not yet been reported. Abbreviations: CPCs, cardiac progenitor cells; MSCs, mesenchymal stem cells; CDCs, cardiosphere-derived cells.

**Table 2:** Exsomal lncRNA and circRNA which are identified to modulate cardiac remodeling

Exsomal cargo	Communication	Targets	Functions	References
lncRNAs				
AK139128	Between cardiomyocytes and cardiac fibroblasts	ND	Promotes cardiac fibroblasts apoptosis and inhibits proliferation, migration, and invasion	75
KLF3-AS1	Between MSCs and cardiomyocytes	miR-138-5p/sirt1	Reduces cardiomyocytes apoptosis	76
MALAT1	Between UMSCs and cardiomyocytes	NF-κB/TNF-α	Prevents aging-induced cardiac dysfunction	77
H19	Between MSCs and endothelial cells	ND	Protects cardiomyocytes by enhancing neovascularization	78
ZFAS1	Between cardiomyocytes and heart	miR-4711-5p/wnt4	Promotes cardiac fibrosis	79
circRNAs				
hsa_circ_0097435	Between plasma and heart	ND	Promotes cardiomyocyte apoptosis	85
circ-0001273	Between UMSCs and cardiomyocytes	ND	Reduces cardiomyocytes apoptosis	86

“ND” (Not Determined) indicates that a target has not yet been reported. Abbreviations: MSCs, mesenchymal stem cells; UMSCs, umbilical cord mesenchymal stem cells.

**Table 3:** Clinical studies of exsomal miRNAs in cardiac remodeling

Exsomal miRNAs	Pathology	Biospecimen	References
miR-1 miR-24 miR-133a miR-133b miR-210	CABG surgery	plasma	90
miR-1 miR-133b miR-208b miR-499	MI	plasma	91
miR-210-3p	atrial fibrillation	atrial myocytes, serum	92
miR-136-5p miR-144-5p miR-624-3p miR-624-5p miR-1284-5p	cardiac sarcoidosis	plasma, serum	93
miR-29a	Obesity related cardiomyopathy	plasma	94

Abbreviations: CABG, coronary artery bypass graft; MI, myocardial infarction.

## Abbreviations

CVDs: cardiovascular diseases
HF: heart failure
ECs: endothelial cells
mRNAs: messenger RNAs
ncRNAs: non-coding RNAs
miRNAs: microRNAs
lncRNAs: long noncoding RNAs
circRNAs: circular noncoding RNAs
ceRNAs: endogenous RNAs
MVBs: multivesicular bodies
ESCRT: endosomal sorting complexes required for transport
SNARE: soluble N-ethylmaleimide-sensitive component attachment protein receptor
MI: myocardial infarction
Ang II: angiotensin II
SORBS2: sorbin and SH3 domain-containing protein 2 (SORBS2)
PDLIM5: PDZ and LIM domain 5
MAPKs: mitogen-activated protein kinases
CPC: cardiac progenitor cells
PDCD4: programmed cell death 4
MSCs: mesenchymal stem cells
CDCs: cardiosphere-derived cells
TGF-β: transforming growth factor-β
EVs: extracellular vesicles
UMSCs: umbilical cord mesenchymal stem cells
